# New tricks of an old molecule: lifespan regulation by p53

**DOI:** 10.1111/j.1474-9726.2006.00228.x

**Published:** 2006-10

**Authors:** Johannes H Bauer, Stephen L Helfand

**Affiliations:** Department of Molecular Biology, Cell Biology and Biochemistry, Division of Biology and Medicine, Brown University, Laboratories for Molecular Medicine Providence, Rhode Island, USA

**Keywords:** p53, Aging, Cancer

## Abstract

As guardian of the genome the tumor suppressor p53 controls a crucial point in protection from cellular damage and response to stressors. Activation of p53 can have beneficial (DNA repair) or detrimental (apoptosis) consequences for individual cells. In either case activation of *p53* is thought to safeguard the organism at large from the deleterious effects of various stresses. Recent data suggest that the function of p53 might also play a role in the regulation of organismal lifespan. Increased p53 activity leads to lifespan shortening in mice, while apparent reduction of p53 activity in flies leads to lifespan extension. Although the mechanism by which p53 regulates lifespan remains to be determined, these findings highlight the possibility that careful manipulation of p53 activity during adult life may result in beneficial effects on healthy lifespan.

It has long been known that mice that are deficient for p53 are prone to develop tumors and die prematurely (Donehower *et al*., 1992). Even one functional copy of the p53 gene can protect mice dramatically from these cancers, albeit not quite as effectively as two functional copies. Therefore, more p53 activity appeared to be more protective against cancers.

In 2002, Donehower and colleagues serendipitously studied the effects of increased p53 activity, when, due to a targeting event gone wrong, they created a mouse that expressed a truncated version of p53 (Tyner *et al*., 2002). This truncated version, p24, is thought to bind to regular length wild-type p53. This unique complex is hypothesized to possess increased constitutive activity (Vijg & Hasty, 2005). As predicted, increased p53 activity was very effective against tumor formation. In the mouse line with the strongest allele only two mice, or 6%, developed cancers. In contrast, 48% of wild-type animals and over 80% of mice with only one functional copy of the tumor suppressor developed cancers. These results were confirmed 2 years later with the generation of a mouse by Scrable and colleagues that over expresses a naturally occurring truncated version of p53 (Maier *et al*., 2004). This p44 molecule functions in a similar way to the artificial short p24 isoform and binds full length p53 to bestow heightened activity to endogenous p53 by forming a heteromeric complex. As with the earlier hyperactive p53 model, these mice are exceptionally resistant to cancer. The hypothesis was further put to the test by Serrano and colleagues who engineered a mouse with an additional copy of the whole p53 genomic region. Once again, these mice were remarkably tumor resistant (Garcia-Cao *et al*., 2002). Similar results were obtained by Perry and colleagues when the activity of the p53 inhibitor Mdm2 was decreased, which boosted inducible p53 activity (Mendrysa *et al*., 2006).

In humans, lifespan gains over the last century have in part been achieved due to better treatment of cancers. Do therefore mice also benefit from improved tumor suppression? Mice, in which only the magnitude of the inducible p53 response had been increased (through extra p53 gene dosage or reduction of Mdm2 activity), surprisingly did not live longer than controls. Even more astonishing, instead of living longer, the mice with constitutively hyperactive p53 (through incorporation of a shortened form of p53 into the p53 tetramer), actually died earlier than their wild-type counterparts.

Why does increased tumor protection not translate into longer lifespans for these animals? Several markers of aging, including muscle loss, osteoporosis, skin atrophy and defects in wound healing, were found to be greatly accelerated in mice with hyperactive p53. No markers of accelerated aging were found in the other two models, leading to the speculation that, rather than increased p53 activity per se, altered functionality of the p44(p24)/p53 complex might explain those premature aging phenotypes (Mendrysa *et al*., 2006). That p53 might play a role in lifespan regulation is further suggested by other mouse models with premature aging phenotypes (Vogel *et al*., 1999; Cao *et al*., 2003). Taken together, these observations indicate that modification of p53 function affects longevity and clearly establish a link between p53, cancer formation and lifespan regulation.

If p53 indeed controls the crossroad between cancer and aging, then tampering with p53 activity could either yield longer lifespan, albeit at the cost of increased cancer risk, when its activity is diminished, or low tumor risk, at the cost of a shortened lifespan, if its activity is increased (Campisi, 2002; Donehower, 2005). Neither of these options are particularly attractive for practical applications. However, postmitotic cells are largely resistant to tumor formation, which might help avoid those detrimental trade-offs. If tumor formation could be avoided, reduction of p53 activity might then be able to extend healthy lifespan. Thus, we decided to test the hypothesis that reduced p53 activity might lead to beneficial effect on lifespan in an organism that consists of mostly postmitotic tissues, the fruit fly *Drosophila melanogaster*. The fly genome contains a fairly well conserved p53 homolog, Dmp53, that has been shown to regulate apoptosis in the developing fly embryo (Nordstrom & Abrams, 2000). Not surprisingly, the lifespan of p53 null flies is shortened compared with their wild-type controls (Bauer *et al*., 2005). This most likely reflects a positive role of p53 during embryonic development (Jassim *et al*., 2003). However, when p53 activity is reduced specifically in neurons through the expression of dominant negative versions of Dmp53, lifespan extension of up to 58% is seen without a loss in fecundity or physical activity. Furthermore, when expression of the dominant negative versions of Dmp53 is restricted to only adult neurons, flies also live longer than controls (up to 26% longer). Expression of these same dominant negative constructs in other adult fly tissues including muscle and fat body yields no lifespan extension, and perhaps even a lifespan shortening. These observations give strong support to the hypothesis that a reduction of p53 activity can have beneficial effects on lifespan if tumor formation can be avoided and furthermore highlight the importance of the nervous system in longevity determination. These findings could then be transferred to mammalian systems, as even mammalian neurons are much more tumor resistant than other dividing tissues.

How does reduction of p53 activity extend lifespan? One clue comes from the observation that calorie-restricted long-lived flies do not show additional lifespan extension when DN-Dmp53 is also expressed, indicating that Dmp53 and calorie restriction are related (Bauer *et al*., 2005). Exposing flies to calorie restriction (CR) has been shown to increase levels of the histone deacetylase dSir2 (Rogina *et al*., 2002) and neuronal overexpression of dSir2 extends *Drosophila* lifespan (Rogina & Helfand, 2004). In addition, CR-dependent lifespan extension is blocked in flies lacking dSir2. Interestingly, it is known in mammalian systems that the dSir2 ortholog SIRT1 can deacetylate and inactivate p53 (Smith, 2002). Taken together, these results suggest that CR may in part be mediated by sirtuins and their downstream target p53.

In contrast, modulation of the insulin-signaling pathway through overexpression of dFOXO only leads to extended lifespans when dFOXO is overexpressed in the fat body, but not when overexpressed in neurons (Giannakou *et al*., 2004; Hwangbo *et al*., 2004). As noted above, this is the opposite of what is seen with expression of dominant negative Dmp53 constructs; neuronal, but not fat body expression is important for lifespan extension. On the surface, these data suggest that the insulin-signaling pathway and the CR/Sir2 pathway are two separate mechanisms for lifespan modulation in the fly. Tissue-specific signaling pathways might exist that respond to different cues and control lifespan by different mechanisms. However, it may be that these two disparate pathways are more related than initially expected. Similar to p53, the activity of FOXO is modified by SIRT1 (Brunet *et al*., 2004; Daitoku *et al*., 2004), and in mammals CR is known to change insulin levels (Wanagat *et al*., 1999). Furthermore, p53 has been linked directly to insulin-signaling (Hursting *et al*., 2001, 2004; Maier *et al*., 2004). Thus, there may be points of interaction or cross-talk between these two pathways, either at an intra- or at an intercellular level. Thus, these two different pathways may turn out to be part of the same pathway. In this scenario, stimulating a particular pathway in one tissue (nervous system or fat body) could result in a signal that is transmitted to the other tissue, inducing systemic changes leading to a prolongation of lifespan. Future experiments will certainly untangle these difficult questions.

Regardless of whether p53 is part of the CR or the insulin pathway, or both, its downstream effectors in the fly remain unknown. In developing embryos, Dmp53 has been shown to control apoptosis, but not cell cycle arrest (Brodsky *et al*., 2000; Jin *et al*., 2000; Ollmann *et al*., 2000), and loss of Dmp53 leads to dysregulation of DNA repair pathways (Sogame *et al*., 2003). It is conceivable that down-regulation of Dmp53 leads to a diminished response to stimuli that would otherwise lead to apoptotic cell death. In the brain, this might account for slow cell loss of neurons that cannot be replaced (Campisi, 2002), thus leading to aging. Induction of apoptosis by Dmp53 has been shown in embryos to be regulated at the transcriptional level through up-regulation of the pro-apoptotic molecule reaper (Brodsky *et al*., 2000). However, this functionality appears to be changed during larval and pupal development, as well as in the adult, as reaper-related Dmp53-responsive transcriptional reporters lose their ability to respond to Dmp53-activating stimuli (Bauer & Helfand, unpublished data). The only well-described functionality of Dmp53 in the fly is the induction of caspase-dependent apoptosis, yet two different studies found no evidence to support a role for neuronal, caspase-dependent apoptosis in longevity determination (Bauer *et al*., 2005; Zheng *et al*., 2005).

If Dmp53 does not regulate caspase-dependent apoptosis in the adult fly brain, what other mechanisms could account for the lifespan extension seen by overexpression of DN-Dmp53? One possibility is other forms of neuronal cell death that may normally play a role in the aging process.

Apoptosis has been demonstrated to proceed by caspase-independent pathways through apoptosis-inducing factor (AIF), but no *Drosophila* homolog of AIF has been described thus far. Autophagy has been shown to be a major contributing factor to neuronal cell death (Yuan *et al*., 2003). While most autophagy in neurons is tightly controlled and appears to be protective against neurodegenerative disorders, dysregulation of autophagy might also result in cell loss. The role of autophagy in aging, however, is poorly understood and remains to be elucidated. Another form of cell death that, like autophagy, is characterized by cytoplasmic vacuoles, has been called paraptosis. Paraptosis may be induced by the insulin-like growth factor receptor and is mediated by mitogen activated protein (MAP) kinases (Broker *et al*., 2005). This might link paraptosis to p53, as mice with hyperactive p53 also show hyperactivation of the insulin-like growth factor (IGF) pathway (Maier *et al*., 2004). The role of paraptosis in aging or neurodegenerative disease has not been determined yet.

Recently, a form of programmed cell death has been described that shares features of both apoptosis and necrosis and has subsequently been termed necroptosis (Degterev *et al*., 2005). Interestingly, necrostatin-1, a compound that blocks necroptosis, was found to be protective against cell death induced by ischemic brain injury in mice. This indicates that some neuronal cell loss occurs by a necroptotic mechanism and suggests a novel pathway that reduction of Dmp53 activity could exploit to protect the brain from cell loss of essential cells. It remains to be seen if necroptotic cell loss occurs in the aging fly brain and if necrostatin-1 can protect against it.

An alternative to losing important functionality in the brain by permanent cell loss would be to functionally disable or alter cells without actually killing them. In fibroblasts, replicative senescence constitutes such a pathway (Campisi, 2005). Importantly, replicative senescence is indeed controlled by p53. Certainly, replicative senescence cannot occur in postmitotic cells such as neurons. However, similarly p53-controlled events could take place that irrevocably change or shut down neuronal function, while keeping the cells themselves alive. Markers for such a disabled state have yet to be developed. Loss of functional neurons, either through a cell death or a senescence-like mechanism, could conceivably contribute to aging phenotypes and be counteracted by reduction of p53 function.

Finally, p53 has been demonstrated to activate apoptosis in a transcription-independent manner through interaction with the pro-apoptotic Bcl-2 family member Bax (Chipuk *et al*., 2004). In addition, p53 has also been shown to directly translocate to the mitochondria where it binds Bcl-2 family members Bak, Bcl-2 and Bcl-XL (Mihara *et al*., 2003; Leu *et al*., 2004). Inside the mitochondria, p53 can bind to manganese-superoxide dismutase, inhibiting its scavenger activity (Zhao *et al*., 2005). In either scenario, a breakdown of mitochondrial membrane potential is the consequence, followed by the release of pro-apoptotic factors and loss of mitochondrial functionality. Either catastrophic breakdown of mitochondrial function or simply a contribution by p53 to chronic mitochondrial dysfunction might be a factor underlying the aging process (Wallace, 2005), which could be offset by p53 reduction.

In light of the fact that Dmp53 transcriptional profile changes during fly development, it might also be possible that Dmp53 function changes, exchanging function as a transcriptional regulator to that of a signaling pathway modulator through direct interaction. It is conceivable that p53 influences biological events other than apoptosis through these direct protein–protein interactions, which might be a major mechanism in the adult fly. Interestingly, two-hybrid analysis has revealed protein interaction partners of Dmp53 that are involved in a variety of processes, including DNA repair and metabolism (Giot *et al*., 2003; Stanyon *et al*., 2004). The role of these interactions in longevity determination has still to be elucidated. One thing, however, is for certain: when scientists thought they had a handle on p53, it has always surprised by revealing a new, unexpected function. The p53 story is far from over.
